# Dental care protocol based on visual supports for children with autism spectrum disorders

**DOI:** 10.4317/medoral.20424

**Published:** 2015-08-04

**Authors:** Maria Grazia Cagetti, Stefano Mastroberardino, Guglielmo Campus, Benedetta Olivari, Raffaella Faggioli, Carlo Lenti, Laura Strohmenger

**Affiliations:** 1 DDS, PhD. Assistant Professor, Dental Clinic University of Milan, WHO Collaborating Centre of Milan for Epidemiology and Community Dentistry, Milan, Italy; 2 DDS, PhD. Dental Clinic University of Milan, WHO Collaborating Centre of Milan for Epidemiology and Community Dentistry, Milan, Italy; 3 DDS, PhD. Assistant professor, Dental Institute, University of Sassari, Italy; WHO Collaborating Centre of Milan for Epidemiology and Community Dentistry; 4 MD. research student Child Neuropsychiatric Clinic, University of Milan, Italy; 5 PsyD, Child Neuropsychiatric Clinic, University of Milan, Italy; 6 MD. Full Professor, Child Neuropsychiatric Clinic, University of Milan, Italy; 7 MD, DDS. Full Professor, Dental Clinic, University of Milan, WHO Collaborating Centre of Milan for Epidemiology and Community Dentistry, Italy

## Abstract

**Background:**

Subjects with Autism Spectrum Disorders (ASDs) have often difficulties to accept dental treatments. The aim of this study is to propose a dental care protocol based on visual supports to facilitate children with ASDs to undergo to oral examination and treatments.

**Material and Methods:**

83 children (age range 6-12 years) with a signed consent form were enrolled; intellectual level, verbal fluency and cooperation grade were evaluated. Children were introduced into a four stages path in order to undergo: an oral examination (stage 1), a professional oral hygiene session (stage 2), sealants (stage 3), and, if necessary, a restorative treatment (stage 4). Each stage came after a visual training, performed by a psychologist (stage 1) and by parents at home (stages 2, 3 and 4). Association between acceptance rates at each stage and gender, intellectual level, verbal fluency and cooperation grade was tested with chi-square test if appropriate.

**Results:**

Seventy-seven (92.8%) subjects overcame both stage 1 and 2. Six (7.2%) refused stage 3 and among the 44 subjects who need restorative treatments, only three refused it. The acceptance rate at each stage was statistically significant associated to the verbal fluency (*p*=0.02; *p*=0.04; *p*=0.01, respectively for stage 1, 3 and 4). In stage 2 all subjects accepted to move to the next stage. The verbal/intellectual/cooperation dummy variable was statistically associated to the acceptance rate (*p*<0.01).

**Conclusions:**

The use of visual supports has shown to be able to facilitate children with ASDs to undergo dental treatments even in non-verbal children with a low intellectual level, underlining that behavioural approach should be used as the first strategy to treat patients with ASDs in dental setting.

**Key words:**Autism spectrum disorders, behaviour management, paediatric dentistry, visual learning methods.

## Introduction

Autism Spectrum Disorders (ASDs) are lifelong neurodevelopmental disabilities and are major psychiatric pathologies in children and adolescents (1). Behavioural intervention is the only well-established approach to treat these disorders (2). Children with ASDs often offer a limited collaboration to medical procedures, particularly those considered invasive as dental care, because they may cause distress and behaviour disturbance (3). Behavioural strategies have been used to teach children with autism to be compliant with medical procedures (4,5) but research on training these children to be compliant with dental procedures is scant (6-9).

ASDs are reported to occur in all racial, ethnic, and socioeconomic groups, yet are on average 4 to 5 times more likely to occur in boys than in girls (1,10). It is estimated that in the United States the average of children with ASDs ranges between 1 in 240 and 1 in 80 (11). A recent study in South Korea reported an average of 3 to 110 children (12).

ADSs include a class of neurodevelopmental disorders characterized by a triad of deficits in social reciprocity, impaired communication and repetitive restricted patterns of behaviour or interests (1). The presence of phobias related to the difficulty of learning, how to deal with new and anxiety-generating visual and auditory stimuli is also reported among children with ASDs. The fear of the dentist is included (6), and parents avoid regular dental examinations and therapies. This habit produces in subjects with ASDs frequently untreated decayed teeth and higher rate of gum inflammation, if compared to individuals without ASDs of the same age group (13,14). In addition, compromised communication often leads to the inability to express discomfort or pain, as those caused by dental disease, in an adequate, prompt and spontaneous way. Moreover it is difficult for the patient to understand the verbal messages that are aimed to soothe and reassure the patient, as well as those aimed to explaining what it will happen during dental care procedures. Finally, compromised communication generates inadequate communication strategies that may turn into problem of behaviours (15). Nevertheless, behavioural attitudes may vary widely in children with ASDs, ranging from collaboration during even bloody procedures to the absolute impossibility in conducting an oral examination (16).

Qualitative compromised interaction, when associated with discomfort, may lead to significant behavioural problems, stereotypes and aggressive behaviours, directed toward others or either self inflicted (17). In addition, unfamiliar environment, as the dental environment and modifications in standard daily routines, as to go to the dentist instead of going to school, often lead individuals with ASDs to appositive behaviours and may generate rage episodes (18). Thus, in order to treat the patient, the dentist faces these situations with the administration of sedatives or providing dental treatment under general anaesthesia (19,20).

Some experiences that use behavioural techniques as a way of reducing anxiety are reported (6,16,21,22). The most common among counter-conditioning procedures is Systematic Desensitization (20). Modeling, as well, has been used before in order to reduce fear in children with typical development, but not in children with ASDs. Even experiences that dealt with positive-negative reinforcement proved merely anecdotal (16,22). Experiences in this field are generally addressed to small numbers of adult patients with mental retardation. Among various researches on children with ASDs that may be regarded as methodologically solid, Luscre and Center (6) should be mentioned: they used a combination of desensitization, video modeling and reinforcement to facilitate dental examination.

Most of the research that is concerned with dental intervention on patients with ASDs involves retrospective studies (14,19).

In 1999 a study was performed to evaluate the use of visual supports to introduce dentistry to children with ASDs (7). Using a picture book describing every step of a dental examination, sixteen children were prepared. Results showed that children were fully cooperative compared to controls. In addition two children from the prepared sample received dental restorations and others two received fissure sealants, while none of the control group accepted any dental treatment. A recent successful study on training adults and children with ASDs to be compliant with a dental examination using a package consisting of behavioural procedure, including visual pedagogy, audiovisual modeling etc. was performed (8). Besides the different behavioural techniques of approach, different sources highlighted the necessity that examinations and treatments in patients with ASDs should last a short amount of time and should involve the minimal amount of sensory stimulation (14).

In San Paolo Hospital, University of Milan, Italy, a strategy was developed to take care of children wit ASDs in the dental setting. The aim of this report is to present a multistage approach based on the use of visual supports to facilitate subjects with ASDs to undergo a oral examination and treatments.

## Material and Methods

- Sample

The study design followed the Declaration of Helsinki and was approved by the Ethics Committee of San Paolo Hospital, Milan Italy (registration number 273).

All patients have been enrolled in San Paolo Hospital - Childhood Neuropsychiatric Clinic, Autism and PDD Diagnosis and Care Centre- from December 2012 to September 2013. A convenience sample of one hundred and two children, who were receiving services from the Centre, was invited to participate. An information leaflet, explaining the aim of the study and requesting their child’s participation with a signed consent form, was given to parents or guardians. 83 subjects with their parent’s signed consent were enrolled, 65 males and 18 females, age range 6-12 years (mean age of 8.7 ± 2.7). Sixty-one children suffered from Primary Autism, 19 from Pervasive Developmental Disorder-Not Otherwise Specified, 2 from Asperger’s Syndrome and 1 from Disintegrative Disorder. The diagnosis has been performed by a child and adolescent psychiatrist and by a psychologist, using the Diagnostic and Statistical Manual of Mental Disorders (fourth edition criteria). The children have resulted to be unaffected by any other neurologic or sensorial deficit. A child psychiatrist (CL) measured the intellectual level of each child using the Wechsler Intelligence Scale for Children (23). Patients with normal intellectual level (QI >70) were 20, those with a mild intellectual disability (QI between 40 and 69) were 21 and finally children with severe/profound intellectual disability (QI <39) were 42. Verbal fluency was evaluated using the Autism Diagnostic Observation Schedule (ADOS) (24). Patients who may speak fluently were 32, those who have developed a non-fluent language (single words or echolalia) were 32 and finally non-verbal patients were 19.

- Visual multistage protocol

None of these subjects, as parents reported, underwent to dental examination before the enrollment.

The multistage visual protocol is divided into four stages, the first three proposed to all children, and the fourth reserved to those subjects with active caries lesions. Different goals were set for each stage: accept oral examination sitting in a dental chair (stage 1); accept professional oral hygiene session (stage 2); accept fissure sealant procedure (stage 3); finally, accept restorative treatment (stage 4).

**Figure 1:**
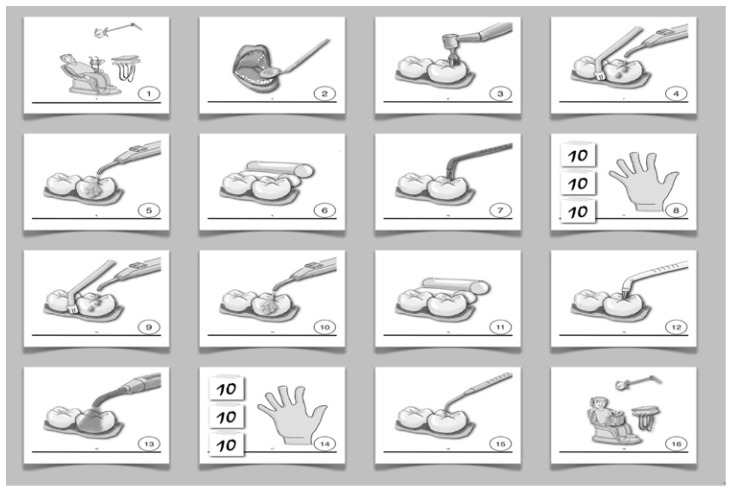
Visual supports used for the training in stage 3 (fissure sealant).

Subjects were facilitated to the four goals through the use of ad hoc prepared visual supports (Fig. 1). A dentist (MGC) made a sketch of each single step of the four procedures (oral examination, dental hygiene session, sealants and restorative treatment) and with the aid of a psychologist (RF), specialized in the treatment of children and adolescents with ASDs, the final visual supports were created. Visual support for oral examination (stage 1) was used by the psychologist (RF) during one hour training, repeated for 8 times (two sessions a week for one month), carried out individually for each child. Finally, the children undergo to the oral examination while a parent showed the child the same supports used by the psychologist for the training. Two dentists (MGC and SM) using the Rud and Kisling Scale (25) evaluated the degree of cooperation offered by the child during the oral examination as cooperative acceptance, indifferent acceptance, reluctant acceptance and nonacceptance.

Subjects who completed stage 1 received a new training by parents at home. Parents received one hour training provided by a dentist (GC) and a psychologist (BO), learning how to use the visual supports for each procedure. Parents were instructed to use the visual supports for at least fifteen minutes a day. After a two weeks training, the child came back to the dental team that tried to perform the procedure, while a parent showed to child the same supports used at home. Each further stage came after a new training, performed at home by parents, using ad hoc prepared visual supports. Only children who have completed each step have been admitted to the next.

- Dental procedures

Each treatment lasted at least 30 minutes. Procedures were performed in order to reduce the amount of sensory stimuli. The light of the dental unit was soft, the suction device was always introduced at low regime to minimize the noise; dental instruments were prepared before the patient entered into the room to minimize confusion and the noise of the broken envelops.

Data analysis

All data were imputed into a spread sheet (Excel Microsoft®). Regarding the age, the sample was divided in three groups (6-7 years, 8-9 years and 10-12 years). Association between acceptance rates at each stage and gender, age group, intellectual level, verbal fluency and cooperation grade was tested with chi-square test if appropriate. All the analyses were carried out using STATA 10 software.

## Results

**Figure 2:**
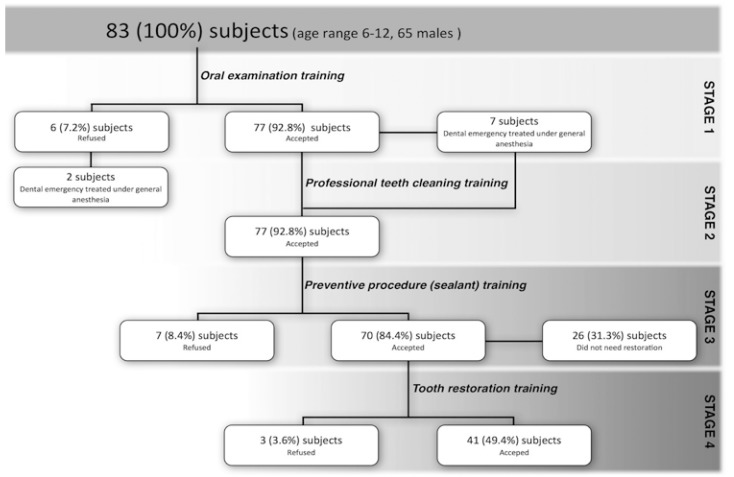
Flow chart of the study design with acceptances and refusals at each stage, expressed as numbers and percentages.

The acceptance rate across the different stages is displayed in figure 2. Seventy-seven subjects (92.8%) overcome both stage 1 and 2, while six subjects (7.2%) refused stage 3. The remaining sample was then divided according to the restorative treatment needs: 44 (53.0%) subjects need some restorative treatment; only three of them refused it.

The acceptance rate at each stage was associated to verbal fluency, intellectual level and degree of cooperation offered by the child during oral examination ( Table 1). Regarding verbal fluency, a statistically significant association was observed at each stage: *p*=0.02; *p*=0.04; *p*=0.01, respectively at stage 1, 3 and 4. At stage 2 all subjects accepted to move to the next stage. According to the intellectual level, a statistically significant association to the acceptance rate was found at stage 4 only (*p*=0.04); while, according to the collaboration grade evaluated by the dental team during the oral examination, a statistically significant association with the acceptance rate was found at stage 1 and 2 (*p*=0.04) and it was slightly near the statistically significance at stages 3 and 4 (*p*=0.07 and 0.06, respectively). Gender and age group were not statistically associated with acceptance rate at any stage (data not in table).

**Table 1:**
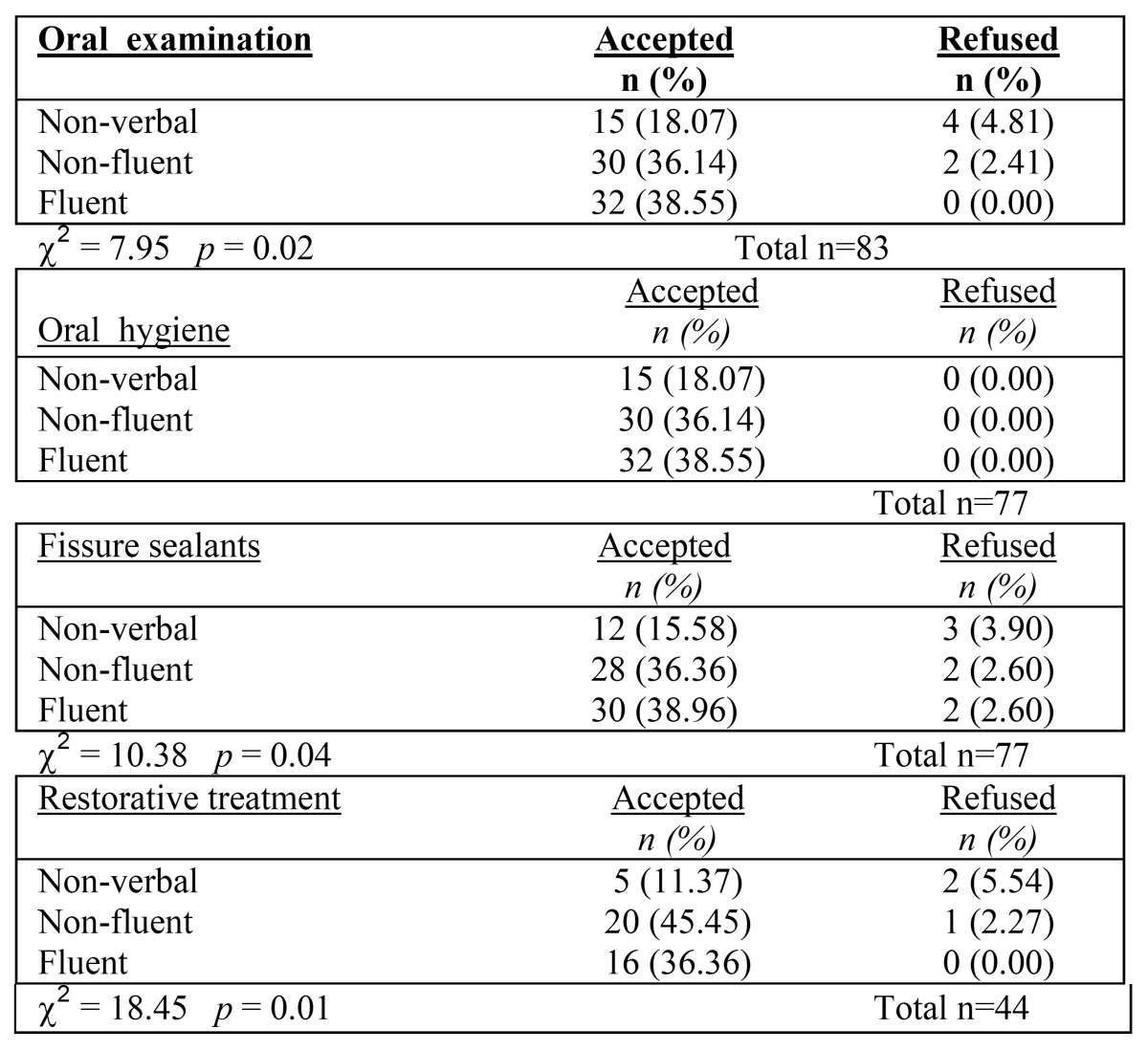
a,b,c. a- Sample distribution according to verbal fluency and acceptance rate for each dental procedure.

Data analysis showed that verbal fluency, intellectual level and grade of cooperation had a confounding effect on the acceptance rate when tested by interaction model (likelihood ratio test statistic, G=6.144 *p*<0.01), so a dummy variable was created as the sum of the three variables. The association between the dummy variable and each stage was always statistically significant; results on fissure sealants (stage 3) are displayed in  Table 2.

**Table 2:**
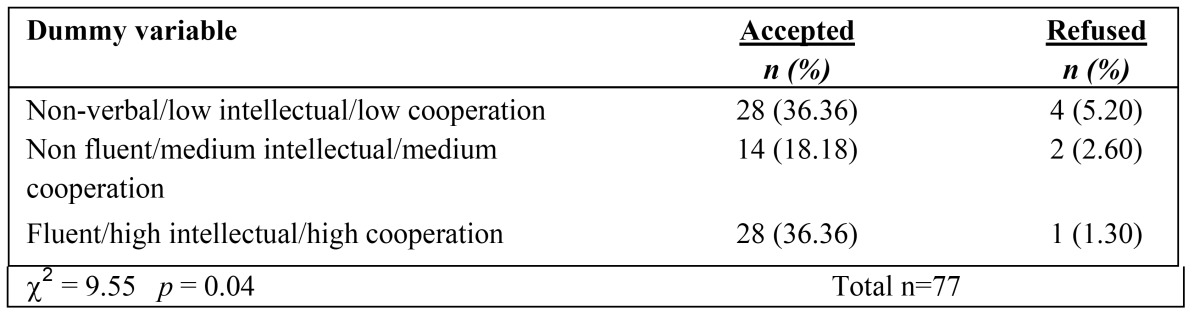
Sample distribution according to the different scores of the dummy variable (sum of verbal fluency, intellectual level and degree of cooperation) and acceptance rate for fissure sealants.

## Discussion

The present report describes a multistage visual protocol for children with ASDs in order to realize an oral examination and treatments. Results show that the use of visual supports may represent a strategy to provide dental care to young subjects with ASDs. A good compliance to dental treatments was observed in the majority of subjects, regardless verbal fluency, intellectual level, age and gender; a professional hygiene procedure was realized in over the ninety per cent of the sample and a fissure sealant was applied in over the eighty per cent of the subjects.

Research on training children with ASDs to be compliant with dental procedures is scant and limited to simple procedure as dental examination (6-9).

Applied Behaviour Analysis (ABA) is a branch of psychology focusing on the analysis and modification of human behaviour; it examines the functional relationship between environment and behaviour to modify social significant behaviour. ABA can be useful for people with ASDs in several settings such health and dental care, as it increases tolerance to medical procedure (14,21). The visual protocol tested in the present report is based on ABA principles and overall the dental treatment should be seen as a team effort focused on identifying problematic behaviour in the dental setting and modifying them.

Subjects with communicative disabilities, as children with ASDs, benefit from structured time and space (26). The use of intensive behaviour–based programming has shown to be an effective strategy for training these patients (27).

It is notorious that visual elaboration is strength for people with ASDs, even for those individuals with a normal intellectual level and fluent speech. People with autism learn with preference or more easily when visual instruments are used, because these supports allow a reduced amount of words and send a message that is constant and steady (28). In addition, recent findings have demonstrated that children with ASDs are able to contextualize pictures and use them to adaptively guide their behaviour in real situations (28). Moreover, in complex procedures, visual support may be useful for breaking down multiple steps facilitating children with ASDs to be in compliance with each step (29). In the present protocol, this approach was tested for the dental setting and the results strengthen this evidence. The visual protocol tested has allowed performing in children with ASDs the most common dental treatments that are usually realized in young subjects, without sedative use or general anesthesia.

Verbal fluency is the ability to generate novel verbal responses. Data from the present report show that refusals to accept dental treatment occurred most frequently in nonverbal children, less in non-fluent ones and even less in those with fluent speech. With regards to the intellectual level, a statistically significant association with the acceptance rate was found for the restorative treatment, the last and more complex stage of the path. According to the collaboration grade evaluated by the dental team during the oral examination, a statistically significant association with the acceptance rate was found for the professional oral hygiene, while for fissure sealant and restorative treatment the acceptance rate was slightly near the statistically significance. Statistically significant results were also found when a dummy variable as the sum of verbal fluency, intellectual level and degree of cooperation was created and compared to the acceptance rate at each stage. These results suggest that the visual supports seem to be more effective in facilitate children with ADSs with higher verbal and intellectual skills; nevertheless the refusals of dental procedures were few also in children with lower skills. In addition, even those subjects judged during the oral examination as offering a low level of cooperation, have frequently overcome the different stages of the path.

At the best of our knowledge, this is the first report showing the use of visual supports to obtain the acceptance of invasive dental procedures as fissure seal ants and restorative treatments in children with ASDs. Another strength of this approach is that the experience of the multistage path represents the achievement of a personal autonomy. This autonomy could probably be transferred, allowing the child to receive more easily other physical examination.

However, some weaknesses are present. First of all, in the present report no control group was used to verify how much the ability of subjects with ASDs to accept treatment was enhanced by visual approach, as shown in similar studies (6-8). It is well known that for subjects with ASDs medical and dental treatments are often extremely difficult to tolerate (6). In a recent study was reported that approximately half of the examined subjects showed escape or avoidance behaviour during the oral assessment (30). Therefore it is mandatory to offer children with ASDs a different approach to dental procedures. The results of the present report show that even the majority of nonverbal children with a low intellectual level and showing a low degree of cooperation at the oral examination were able to undergo to a dental sealant procedure, goal of the entire sample.

The role of parents in the protocol was crucial. Parents are the most important resource in promoting changes in child’s behaviour (18), supporting health professionals. In this visual multistage protocol, in three of the four stages, the parents have performed the training on their children at home, after they have been trained them self. Parents’ cooperation would represent a limit, if they are not able to carry on the training due to lack of time or capacity. Nevertheless, all parents took part in the multistage visual approach with enthusiasm and collaborative spirit. They demonstrated to have understood the importance of a healthy mouth for their children; moreover this result could be a new milestone in children’s life and parents willing to achieve it.

This report underlines that behavioural intervention should be used as the first strategy to treat patients with ASDs in dental setting.
